# Inverse correlation between *TP53* gene status and PD-L1 protein levels in a melanoma cell model depends on an IRF1/SOX10 regulatory axis

**DOI:** 10.1186/s11658-024-00637-y

**Published:** 2024-09-05

**Authors:** Lucia Martinkova, Pavlina Zatloukalova, Martina Kucerikova, Nela Friedlova, Zuzana Tylichova, Filip Zavadil-Kokas, Ted Robert Hupp, Philip John Coates, Borivoj Vojtesek

**Affiliations:** 1 RECAMO, Masaryk Memorial Cancer Institute, 602 00 Brno, Czech Republic; 2 National Centre for Biomolecular Research, Faculty of Science, Masaryk University, 625 00 Brno, Czech Republic; 3 Department of Experimental Biology, Faculty of Science, Masaryk University, 625 00 Brno, Czech Republic; 4 Institute of Genetics and Molecular Medicine, University of Edinburgh, Edinburgh, Scotland EH4 2XR UK

**Keywords:** IFNγ, IRF1, PD-L1, p53, SOX10

## Abstract

**Background:**

PD-L1 expression on cancer cells is an important mechanism of tumor immune escape, and immunotherapy targeting the PD-L1/PD1 interaction is a common treatment option for patients with melanoma. However, many patients do not respond to treatment and novel predictors of response are emerging. One suggested modifier of PD-L1 is the p53 pathway, although the relationship of p53 pathway function and activation is poorly understood.

**Methods:**

The study was performed on human melanoma cell lines with various p53 status. We investigated PD-L1 and proteins involved in IFNγ signaling by immunoblotting and mRNA expression, as well as membrane expression of PD-L1 by flow cytometry. We evaluated differences in the ability of NK cells to recognize and kill target tumor cells on the basis of p53 status. We also investigated the influence of proteasomal degradation and protein half-life, IFNγ signaling and p53 activation on biological outcomes, and performed bioinformatic analysis using available data for melanoma cell lines and melanoma patients.

**Results:**

We demonstrate that p53 status changes the level of membrane and total PD-L1 protein through IRF1 regulation and show that p53 loss influences the recently discovered SOX10/IRF1 regulatory axis. Bioinformatic analysis identified a dependency of SOX10 on p53 status in melanoma, and a co-regulation of immune signaling by both transcription factors. However, IRF1/PD-L1 regulation by p53 activation revealed complicated regulatory mechanisms that alter *IRF1* mRNA but not protein levels. IFNγ activation revealed no dramatic differences based on *TP53* status, although dual p53 activation and IFNγ treatment confirmed a complex regulatory loop between p53 and the IRF1/PD-L1 axis.

**Conclusions:**

We show that p53 loss influences the level of PD-L1 through IRF1 and SOX10 in an isogenic melanoma cell model, and that p53 loss affects NK-cell cytotoxicity toward tumor cells. Moreover, activation of p53 by MDM2 inhibition has a complex effect on IRF1/PD-L1 activation. These findings indicate that evaluation of p53 status in patients with melanoma will be important for predicting the response to PD-L1 monotherapy and/or dual treatments where p53 pathways participate in the overall response.

**Graphical Abstracts:**

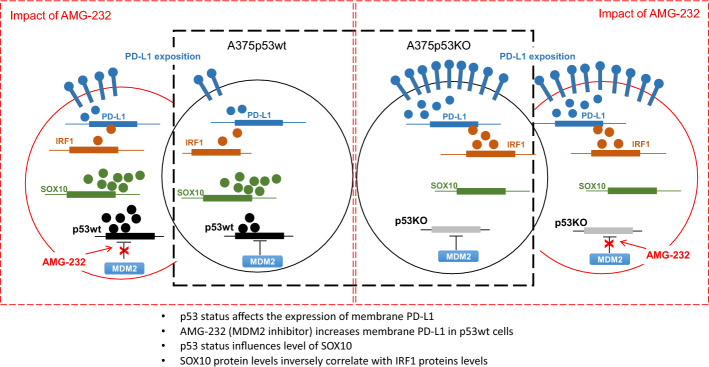

**Supplementary Information:**

The online version contains supplementary material available at 10.1186/s11658-024-00637-y.

## Introduction

Cutaneous melanoma is a highly metastatic tumor type with poor prognosis after metastasis occurs. Current treatment options are based on targeted therapy for *BRAF* mutated tumors and immunotherapy consisting of anti-PD1 (programed cell death 1)/PD-L1 (programmed death ligand 1) and anti-CTLA4 (cytotoxic T-lymphocyte-associated protein 4) checkpoint inhibition [1–3]. Although anti-PD1/PD-L1 immunotherapy in advanced melanoma has shown promising results, many patients are completely refractory or do not show long-lasting remission [4, 5]. Several approaches have been explored for patient stratification for PD1/PD-L1 therapy. It was shown that higher levels of PD-L1 in tumor biopsies correlates with better response to PD-1 immunotherapy [6–10]. Further analysis revealed that PD-L1 does not have sufficient predictive value, and other approaches were introduced, such as determination of mutational burden, mismatch repair deficiency, neoantigen immunogenicity, tumor microenvironment, infiltrating tumor cells, HLA heterozygosity, MHC-I mutations, autoimmune susceptibility, or the gut microbiome [11, 12]. From these studies, intact antigen presentation and IFNγ (interferon-γ) signaling are crucial in the response to immunotherapies [13].

p53 is now realized as an important part of the immune response to tumors via multiple mechanisms, including the antigen presentation machinery [14–16], cytokine production [17, 18] and immune cell infiltration [19, 20], and loss of p53 activity affects immune signaling in both tumor and immune cells [21]. The major negative regulator of p53 is mouse double minute 2 homolog (MDM2), which maintains p53 protein at a low level. After stress, the MDM2-p53 interaction is disrupted, and p53 increases rapidly and transcriptionally upregulates *MDM2* in a negative feedback loop [22]. Several studies have demonstrated the beneficial effect of combining p53 activation by MDM2 inhibitors with PD1/PD-L1 immunotherapy [16, 19, 23, 24], illustrated by enhanced T-cell infiltration and T-cell mediated killing, and activation of IFN I and IFN III pathways. p53 activation by MDM2 inhibitors increases membrane PD-L1, and surprisingly, p53 loss also causes an increase in PD-L1 levels [25]. The mutational status of *TP53* has been shown to correlate with PD-L1 levels in multiple cancer types [26–29]. In melanoma, *TP53* mutation correlates with higher mRNA levels of *CD274* (the gene that encodes PD-L1) and concomitantly higher PD-L1 protein levels. Moreover, *TP53* status seems to impact PD-L1 induction by IFNγ via the Janus kinase/signal transducers and activators of transcription (JAK-STAT) pathway [30].

IFNγ is a major activator of JAK-STAT signaling, leading to direct upregulation of interferon regulatory factor 1 (IRF1), which transcriptionally activates *CD274* [31]. Recently, JAK-STAT independent regulation of IRF1 was described through interferon regulatory factor 4 (IRF4), which is a negative regulator of IRF1 that is transcriptionally regulated by SRY-box transcription factor 10 (SOX10) [32]. Importantly, SOX10 enhances melanocytic development and melanoma cell growth, and SOX10 deficiency is associated with an invasive slow cycling state resulting in acquired resistance to targeted therapies [33, 34].

Here, we investigated the p53 pathway and its impact on IFNγ signaling in regulating PD-L1 levels. Using melanoma cell lines bearing wild-type (wt) p53, p53 knock-out derivatives, and p53-null cell lines cells bearing mutant p53, we investigated the impact of an MDM2 inhibitor on *CD274* mRNA levels, total PD-L1, and membrane PD-L1 protein, and performed natural killer (NK)-cell mediated killing assays to evaluate the effect on the tumor immune response. We identified changes in *IRF1* mRNA and protein levels on the basis of p53 status and activation, and found that SOX10 was also changed on the basis of p53 loss. Analysis of melanoma cell lines and patient samples identified crosstalk between these two pathways and their effects on immune signaling. Despite an increase in PD-L1 in p53-null cells, we also show reduced tumor cell killing in p53-null backgrounds. These findings provide the basis for further research into additional targeted therapies for melanoma patients. Moreover, p53 activation in IFNγ treated cells enhanced *IRF1* mRNA levels and PD-L1 membrane levels, suggesting a novel approach to improve the efficacy of immunotherapy in patients with melanoma.

## Materials and methods

### Cell culture and reagents

A375 (ATCC CRL-1619) cells, *TP53*-null derivatives of A375 (A375p53KO) (described in [25]), SK-MEL2 (ATCC HTB-68), SK-MEL-28 (ATCC HTB-72), SK-MEL-5 (ATTC HTB-70), and RPMI-7951 (ATTC HTB-66) were grown in DMEM. HT-144 (ATCC HTB-63), G-361 (ATCC CRL-1424), MALME-3 M (ATCC HTB-64), and SK-MEL-3 (ATTC HTB-69) were grown in McCoy’s 5A Medium. p53 status and oncogenic mutations in these cell lines are indicated in Table S1. The media used for cell cultivation contained 10% fetal bovine serum (FBS), 1% pyruvate, and penicillin/streptomycin, and all cell lines were incubated at 37 °C in a humidified atmosphere with 5% CO_2_. Cells were grown to 60–80% confluence prior to treatment with the MDM2 inhibitors AMG-232 (Axon Medchem), IFNγ (Thermo Fisher), the proteasome inhibitor MG-132 (Selleckchem), or the protein synthesis inhibitor cycloheximide (CHX) (Calbiochem). SOX-10 knock-out cell lines were prepared by lipofection of Cas9 protein (Thermo Fisher) with sgRNA for *SOX10* (TrueGuide™ Synthetic sgRNA, nucleotide sequence: AAAGCAAGCCGCACGTCAAG, Thermo Fisher) and confirmed by western blotting (WB). Transient transfection was performed using polyethylenimine (Sigma Aldrich) and 500 ng pCDNA3 plasmids expressing full-length wt p53 protein or pCDNA3 empty vector. Cells were harvested 24 h or 48 h post-transfection.

### Western blotting

Cells were harvested into 1X LDS buffer, followed by measurement of protein concentration by DC assay (Bio-Rad). SDS–polyacrylamide gel electrophoresis and immunoblotting were performed as described previously [35]. The antibodies used in this study are listed in Table S2. Chemiluminiscent signals were developed using ECL (Thermo Fisher) and visualized with ChemiDoc imaging system (Bio-Rad). Some membranes were then stripped using 100 mM 2-mercaptoethanol, 2% (w/v) SDS, 62.5 mM Tris–HCl, pH 6.7 for 4 h before re-probing the same blot with antibodies to a different target protein; these blots are indicated by arrows in the figures.

### Reverse transcription quantitative polymerase chain reaction (RT-qPCR)

Total RNA was extracted using RNeasy (Qiagen), and reverse transcribed using SuperScript^™^ IV VILO^™^ Master Mix (Invitrogen). PowerUp^™^ SYBR^™^ Green Master Mix (Thermo Fisher) was used for qPCR. β-actin (*ACTB*) served as endogenous control. The data represent means of technical triplicates within each independent biological replicate. Relative mRNA levels were calculated using the 2^−ΔΔCT^ method. Primer sequences used in this study are: *ACTB*: 5´- GCCGACAGGATGCAGAAGGAG -3´ (sense) and 5´- CTAGAAGCATTTGCGGTGGAC -3´ (antisense), *CD274*: 5´- TACTGGCATTTGCTGAACGC -3´ (sense) and 5´- CTTGTAGTCGGCACCACCAT -3´ (antisense), *IRF1-F*: 5´- ACCCTGGCTAGAGATGCAGA -3´ (sense) and 5´- GCTTTGTATCGGCCTGTGTG -3´ (antisense). Statistical significance was calculated using two-tailed Student’s *t*-test.

### Cell viability assay

Cell viability was determined using Resazurin sodium salt (Sigma-Aldrich). Cells were seeded into 96-well plates (5000 cells per well) and left to adhere for 24 h before treatment with AMG-232 (20–2500 nM) or IFNγ (100 ng/ml). Resazurin (100 mg/l) was added 72 h later and incubated for 3 h at 37 °C. Fluorescence was measured at 530 nm excitation and 590 nm emission using a microplate reader (Tecan Infinite M1000 Pro). Each sample was measured in six technical replicates.

### Flow cytometry

Cells were harvested using Accutase (Merck KGaA), centrifuged at 1000 rpm for 5 min, resuspended in 1% BSA in PBS and kept on ice for 30 min. Cells were centrifuged as before, resuspended in 100 μl allophycocyanin (APC)-conjugated CD274/PD-L1 antibody (Invitrogen) diluted in PBS to 1:200, and incubated for 1 h on ice. After washing in PBS, cells were resuspended in 200 μl PBS, measured on FACSVerse (BD Biosciences), and data were analyzed using FACSuite software (BD Biosciences). PD-L1 on the cell surface was measured as median APC fluorescence intensity, and three independent experiments were performed. Statistical significance was calculated using two-tailed Student’s *t*-test.

### NK cell cytotoxicity assay

The NK-92 cell line (ATCC CRL-2407) was grown in Alpha Minimum Essential medium without ribonucleosides and deoxyribonucleosides (Gibco), supplemented with 0.2 mM inositol (Sigma Aldrich), 0.1 mM 2-mercaptoethanol (Gibco), 0.02 mM folic acid (Sigma Aldrich), 200 U/ml recombinant human IL-2 (Gibco), and 25% FBS at 37 °C with 5% CO_2_. The cells were passaged regularly to maintain a concentration of 2–3 × 10^5^ viable cells/ml.

A375 and A375p53KO cells were stained with 2 μM carboxyfluorescein succinimidyl ester (CFSE) (BD Horizon^™^) in PBS for 10 min at 37 °C. Excess dye was removed by washing once in PBS and twice in DMEM. Cells were seeded into 24-well plates (40,000 cells per well). For NK-mediated cell killing assays, cells were treated with IFNγ (100 ng/ml) and AMG-232 (1 µM) 4 h after seeding. After 16 h incubation at 37 °C, CFSE-positive tumor cells (CFSE+) were washed thoroughly to remove treatment substances before co-culture with NK-92 cells in appropriate ratios for 4 h at 37 °C. Tumor cells without NK-92 cells were used as controls. Culture medium and adherent cells were harvested, and 7-aminoactinomycin D (7-AAD) (Invitrogen, 5 μl per 100 μl sample) was added 10 min prior to measurement on FACSVerse (BD Biosciences). Data were analyzed using FACSuite software (BD Biosciences). The gating strategy is described in more detail in Fig. S1. The percentage of dead tumor cells (CFSE+ , 7-AAD+) was obtained after subtracting the percentage of dead cells in the respective tumor cell only control sample. Statistical significance was calculated using two-tailed Student’s *t*-test.

### Co-expression analysis

Co-expression analysis was performed using cbioportal.com [36, 37]. The data for *TP53*, *SOX10*, and *IRF1* were downloaded from the Skin Cutaneous Melanoma (TCGA, Firehose Legacy), comprising 488 samples. Genes showing statistically significantly co-expression (P < 0.01) were analyzed. Venn diagrams were generated by VennDiagram R library (VennDiagram: Generate High-Resolution Venn and Euler Plots. R package version 1.7.3.; https://CRAN.R-project.org/package=VennDiagram). Co-expressed genes were searched for pathway enrichment using KEGG (Kyoto Encyclopedia of Genes and Genomes) pathway database [38] and visualized using ShinyGO 0.77 [39]. Analysis based on *TP53* status was performed on the SKCM data pack (472 samples, listed as cohort GDC TCGA Melanoma) obtained from cbioportal [36, 37]. Samples were divided into three groups on the basis of *TP53* status (without mutation, with mutation, and hot-spot mutation). These groups were compared with mRNA levels using DESeq2 [40] and dplyr R-packages (dplyr: A Grammar of Data Manipulation. R Package Version 0.4.3.http://CRAN.R-project.org/package=dplyr).

### Analysis of data from DepMap

*SOX10* gene dependency was analyzed using data from the Cancer Dependency Map project (DepMap) [41, 42] based on CRISPR screens (DepMap Public 23Q2 + Score, Chronos release, extracted 23 October 2023). The 100 most co-dependent genes were searched for p53 pathway genes and the co-dependency scores (Pearson and Spearman correlation coefficients) with *P*-values shown in Fig. 4A. The Chronos score was plotted to *TP53* damaging mutation status using all cell lines (https://depmap.org/portal/interactive/?filter=&regressionLine=false&associationTable=false&x=slice%2FChronos_Combined%2F35752%2Fentity_id&y=slice%2Fmutations_damaging%2F38037%2Fentity_id&color =) and using melanoma cell lines https://depmap.org/portal/interactive/?filter=slice%2Fcontext%2FMelanoma%2Flabel&regressionLine=false&associationTable=false&x=slice%2FChronos_Combined%2F35752%2Fentity_id&y=slice%2Fmutations_damaging%2F38037%2Fentity_id&color = .

## Results

### p53 status and activation inversely correlate with PD-L1 in melanoma cell lines

**Fig. 1:**
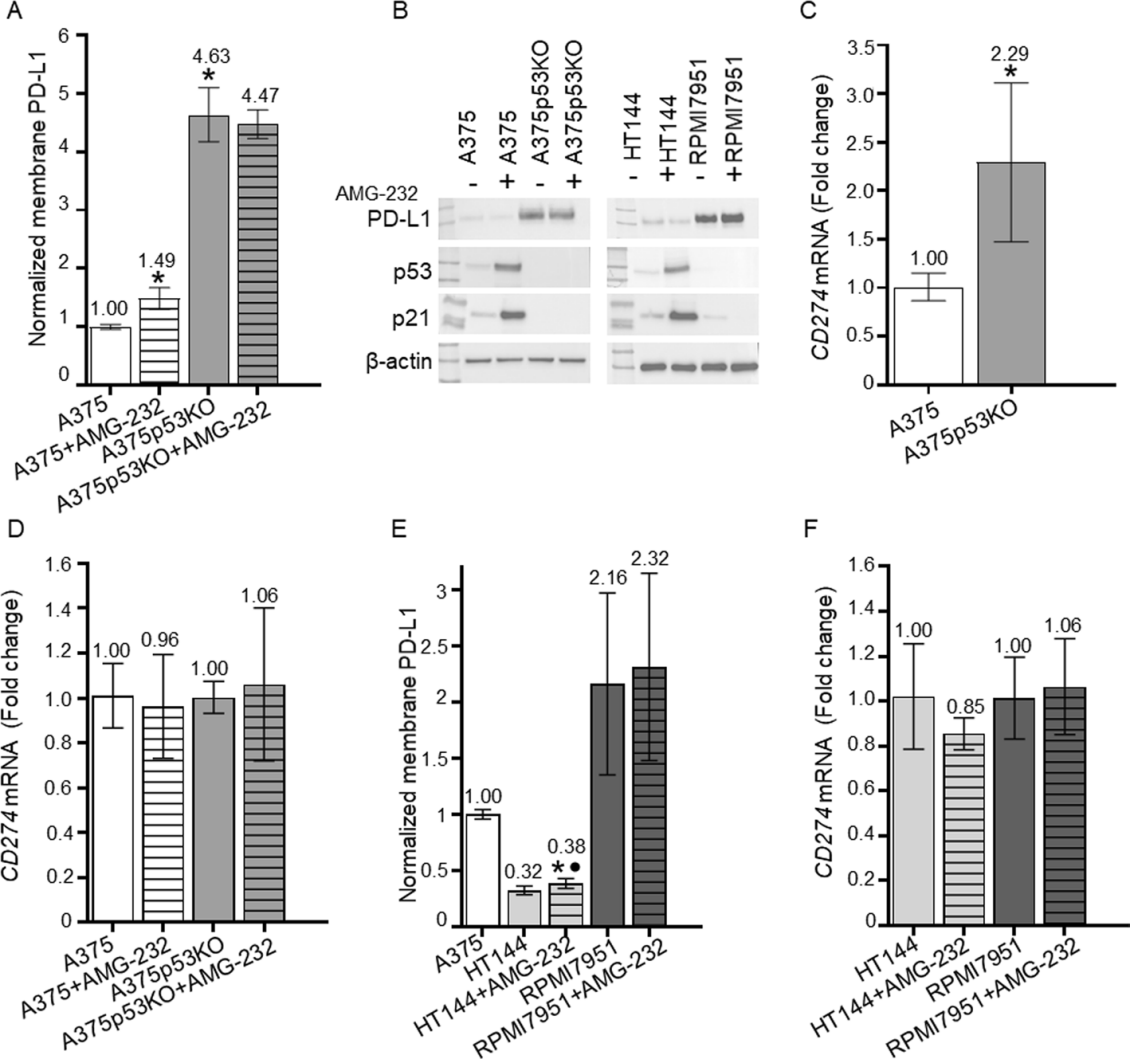
Impact of p53 status and activation on PD-L1 level in melanoma cell lines. **A** Flow cytometric analysis of membrane PD-L1 in wt p53 (A375, white column) and p53-null (A375p53KO, grey column) cells untreated (empty columns) or treated with 1 μM AMG-232 (striped columns) for 24 h. PD-L1 levels were normalized to A375 and are presented as the mean fluorescence intensity of replicates ± SD (*n* = 3) (*P < 0.01). **B** Western blotting in A375, A375p53KO, HT-144 (wt p53), and RPMI-7951 (p53-null) cells treated with or without 1 μM AMG-232 for 24 h. PD-L1, p53, p21, and β-actin (as loading control) have apparent molecular weights of 55, 53, 21, and 42 kDa, respectively. **C** RT-qPCR of *CD274* in A375 and A375p53KO cells, and **D** in A375 (white columns) and A375p53KO (grey columns) cells untreated (empty columns) or treated with 1 μM AMG-232 for 24 h (striped columns). *CD274* mRNA levels were normalized to *ACTB* mRNA (*n* = 3 biological replicates, each with technical triplicates). Relative mRNA levels are shown as fold change ± SEM for A375 compared with A375p53KO (*P < 0.01). **E** Flow cytometric analysis of membrane PD-L1 in A375 and HT-144 (wt p53, white and light grey columns, respectively) and RPMI7951 (p53-null, dark grey columns) cells untreated (empty columns) or treated with 1 μM AMG-232 (striped columns) for 24 h. PD-L1 levels are presented as the mean fluorescence intensity of replicates ± SD (*n* = 3). PD-L1 levels were normalized to untreated A375 (*P < 0.01) or to untreated HT144 cells (*P* < 0.01). **F** RT-qPCR of *CD274* mRNA levels in HT144 (wt p53, light grey columns) and RPMI7951 (p53-null, dark grey columns) cells untreated (empty columns) or treated 1 μM AMG-232 (striped columns) for 24 h. *CD274* mRNA levels were normalized to *ACTB* mRNA (*n* = 3 biological replicates, each with technical triplicates)

PD-L1 protein levels have been reported to vary depending on *TP53* status, with lower levels in wt p53 melanoma cell lines compared with isogenic p53-null derivatives [25, 30]. We validated these observations in melanoma cells with different p53 status, and confirmed that p53KO from A375 cells with wt p53 causes a four- to fivefold increase in membrane PD-L1 (P < 0.01) (Fig. 1A, Fig. S2) and total PD-L1 protein levels (Fig. 1B). RT-qPCR revealed that the increase in PD-L1 protein correlated with a more than twofold increase in *CD274* mRNA levels in cells that lack p53 (P < 0.01) (Fig. 1C), suggesting transcriptional up-regulation induced by p53 loss. To study the effect of p53 activation, we used the MDM2 inhibitor AMG-232 (alternatively KRT-232, navtemadlin) [43], which is currently undergoing clinical trials. This inhibitor binds to the p53-binding pocket of MDM2 and thereby disables p53 proteasomal degradation.

AMG-232 treatment increased membrane PD-L1 levels in parental A375 cells but not in A375p53KO cells (Fig. 1A), in agreement with previous results using nutlin-3a (first-in-class MDM2 inhibitor) [25]. However, p53 activation did not increase total PD-L1 protein levels (Fig. 1B), or *CD274* mRNA in these cells (Fig. 1D), indicating that the total pool of PD-L1 protein is not perturbed by wt p53 activation in these cells. These data indicate that the p53-mediated regulation of membrane localized PD-L1 protein is variable; upon p53 loss, both membrane and total PD-L1 levels are increased, coupled with an increase in *CD274* mRNA; upon pharmacological activation of wt p53, membrane PD-L1 is increased independently of the total protein level or mRNA changes.

We also determined PD-L1 protein levels in two other melanoma cell lines, HT144 with wt p53, and RPMI7951 bearing mutations resulting in a premature stop codon (S166*). p53 is not detected in RPMI7951 cells by WB using the p53 N-terminal antibody DO-1, showing that the protein is not present, whilst HT144 cells show low levels of p53 that are increased after p53 activation, as expected (Fig. 1B). We identified the same pattern of cell membrane PD-L1 and *CD274* mRNA levels in these cell lines. In HT144 with wt p53, the low membrane PD-L1 levels are increased by AMG-232 (*P* < 0.01) (Fig. 1E) without an increase of total PD-L1 protein (Fig. 1B, Fig. S3) or *CD274* mRNA (Fig. 1F). RPMI7951 cells that lack p53 show higher membrane PD-L1 levels that are not increased by AMG-232 (Fig. 1E). These data independently confirm the impact of *TP53* status on basal PD-L1 protein and *CD274* mRNA, and that wt p53 activation affects only membrane PD-L1 levels.

### p53 impacts IFNγ signaling on multiple sites

**Fig. 2:**
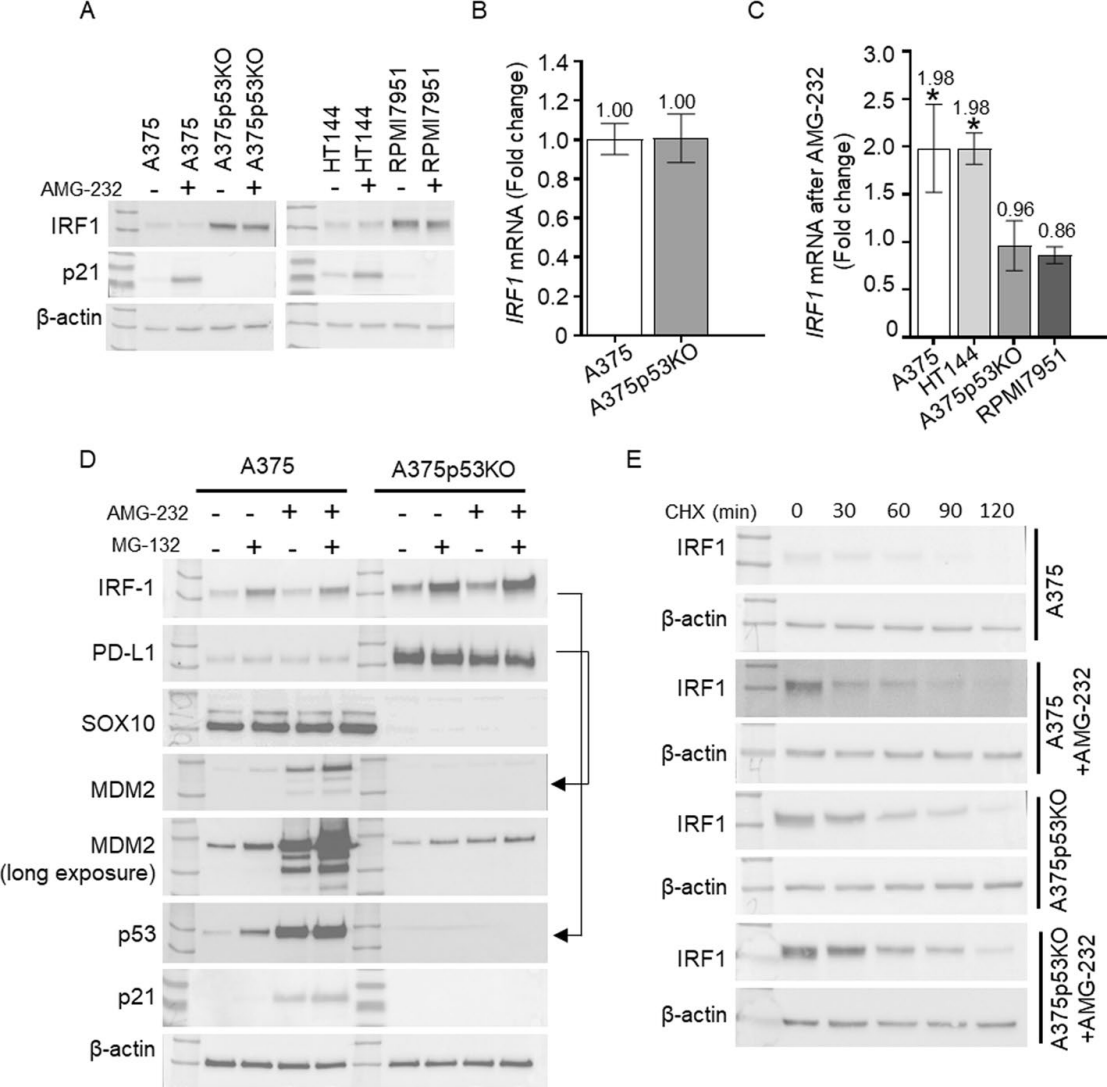
p53 and IFNγ signaling. **A** Western blots in A375 and HT144 (wt p53), and A375p53KO and RPMI7951 (p53-null) cells with or without 1 μM AMG-232 for 24 h. IRF1, p21, and β-actin (as loading control) have apparent molecular weights of 55, 21, and 42 kDa, respectively. **B**, **C** RT-qPCR of *IRF 1* in A375 and A375p53KO cells (**B**) and in wt p53 (A375 and HT144) and p53-null (A375p53KO and RPMI7951) treated with 1 μM AMG-232 for 24 h (**C**). *IRF1* mRNA levels were normalized to *ACTB* mRNA (*n* = 3 biological replicates, each with technical triplicates). Relative expression is shown as fold change ± SEM for treated cells compared with untreated (*P < 0.01). **D** Western blots in A375 and A375p53KO cells with or without 1 μM AMG-232 for 24 h and 20 μM MG-132 for the last 4 h before harvesting. IRF1, PD-L1, SOX10, MDM2, p53, p21, and β-actin (as loading control) have apparent molecular weights of 55, 55, 55, 90, 53, 21, and 42 kDa, respectively. Stripped and re-probed blots are indicated by arrows. **E** Western blots in A375 and A375p53KO cells treated with or without 1 μM AMG-232 for 24 h and with CHX (100 μg/ml) for the indicated times. IRF1 and β-actin (as loading control) have apparent molecular weights of 55 and 42 kDa, respectively

To gain more insight into PD-L1 regulation by p53, we examined the levels of proteins associated with IFNγ signaling through the JAK-STAT pathway that regulates IRF1/PD-L1 (Fig. 2). IRF1 is a direct transcriptional regulator of PD-L1. WB of A375, A375p53KO, HT144, and RPMI7951 cells revealed a clear dependence of IRF1 on p53 status. In wt p53 cells, IRF1 protein levels were low, while a significant increase in IRF1 protein (Fig. 2A), but not mRNA (Fig. 2B), was detected in p53-null cells. Activation of p53 by AMG-232 did not change IRF1 protein levels (Fig. 2A), but *IRF1* mRNA levels were increased in wt p53 cells (Fig. 2C), suggesting that p53 activation can act directly or indirectly as a transcriptional activator of IRF1, and that protein synthesis or degradation mechanisms are also involved.

To understand the mechanisms of IRF1 regulation by p53 status and its activation in more detail, we investigated IRF1 protein half-life and its susceptibility to proteasomal degradation. Proteasome inhibition of A375 and A375p53KO cells increased IRF1 protein levels in the presence or absence of AMG-232, confirming that it is degraded via the proteasome (Fig. 2D). However, AMG-232 treatment had no additional effect on its accumulation. PD-L1 did not accumulate after proteasome inhibition, suggesting other degradation mechanisms. Even the expected transcriptional increase of PD-L1 by stabilized IRF1 is not visible. This may be because MG-132 stabilizes not only IRF1, but also other regulatory proteins, which may contribute to the degradation of PD-L1 through mechanisms other than the proteasome. We next used CHX to inhibit protein synthesis and measured IRF1 protein in four different conditions: normal (basal), in p53-null cells, and in both conditions after p53 activation (Fig. 2E). Additional results showing the combined effect of MG-132 and CHX are provided in Fig. S4. A gradual decrease in IRF1 protein levels was observed, with no significant differences between conditions. These results suggest that there is no effect of MDM2 inhibition on IRF1 protein half-life and degradation, but these can be masked by feed-back loop regulation, in which MDM2 increases upon p53 activation (Fig. 2D). Moreover, MDM2 was shown to be a binding partner of IRF1 participating in its ubiquitination, which may result not only in its degradation, but also in stabilization/regulation of activity depending on specific conditions [44, 45]. However, direct binding of these proteins has not been shown yet for endogenous proteins.

We also investigated JAK1 and JAK2 protein levels as these proteins were suggested to play a key role in p53/PD-L1 regulation [30], but did not see differences in the basal levels of these two proteins in wt p53 compared with p53-null cell lines (Fig. S5). Furthermore, IRF1 can be regulated by STAT1 or nuclear factor kappa-light-chain-enhancer of activated B cells (NFκB), and it has been demonstrated that nutlin-3 inhibits NFκB [46–50]. Thus, we measured total levels and activating phosphorylations of these proteins (P-STAT1-Y701, P-NFkB-S536) but there were no changes after p53 activation or any dependence on p53 status (Fig. S5). These proteins are therefore not involved in the regulatory network influenced by p53 in these cell lines.

These results demonstrate dependence of p53 status on IRF1 protein levels but not JAK1, JAK2, STAT1, and NFκB in these cell lines. Moreover, p53 activation leads to an increase in *IRF1* mRNA, but this does not lead to increased IRF1 protein levels. Altogether, these results show the independent effects of p53 status and p53 activation on IFNγ signaling, and underline the complexity of p53 responses in this pathway.

### SOX10 dependence on *TP53* status and its impact on IRF1

**Fig. 3:**
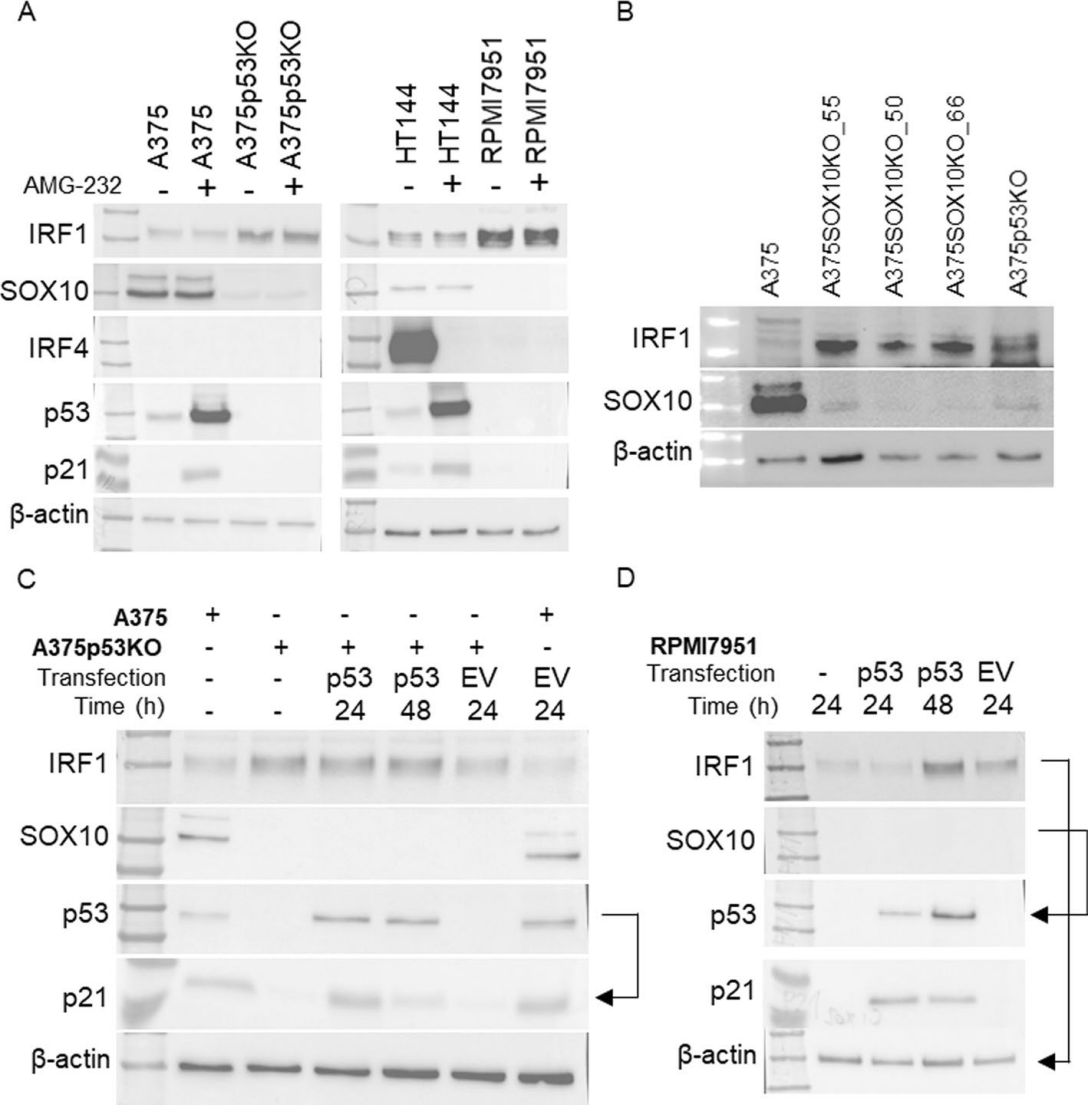
SOX10 dependence on *TP53* status and its impact on IRF1. **A** Western blots in A375 and HT144 (wt p53), and A375p53KO and RPMI7951 (p53-null) cells with or without 1 μM AMG-232 for 24 h. IRF1, SOX10, IRF4, p53, p21, and β-actin (as loading control) have apparent molecular weights of 55, 55, 55, 53, 21, and 42 kDa, respectively. **B** Western blots in A375, A375p53KO, and single cell A375 derivatives with SOX10 deletion (A375SOX10KO). IRF1, SOX10, and β-actin (as loading control) have apparent molecular weights of 55, 55, and 42 kDa, respectively. **C**, **D** Western blots of the indicated proteins in (**C**) A375 and A375p53KO, and (**D**) in RPMI7951 (p53-null) cells transfected with pCDNA3-p53 (p53) or empty vector (EV) for 24 h and 48 h. IRF1, SOX10, p53, p21, and β-actin (as loading control) have apparent molecular weights of 55, 55, 53, 21, and 42 kDa, respectively. Stripped and re-probed blots are indicated by arrows

Recent findings suggest that IRF1 levels are dependent on regulation by SOX10 and IRF4 [32]. SOX10 is a transcription factor for IRF4 that in turn inhibits IRF1 activity [32]. Therefore, we investigated the connections between IRF1 and its regulation by SOX10 and IRF4, and found that IRF1 protein levels inversely correlate with SOX10 proteins levels. SOX10 protein levels are lower in isogenic p53-null cells than the corresponding wt p53 cells (Fig. 3A). Activation of p53 had no effect on SOX10 protein levels, suggesting this regulation is not dependent on p53 transcriptional activity (Fig. 3A). SOX10 has been reported to regulate IRF1 protein levels through IRF4 [32]. We were unable to detect IRF4 by WB in our cell lines, except for HT144 cells (Fig. 3A). Interestingly, activation of the p53 response in this cell line caused a decrease in IRF4 protein. From our data, it seems that IRF4 is not playing a crucial role in SOX10-IRF1 regulation, and another mechanism of regulation is likely involved.

To support or refute the connection between SOX10 and IRF1, we developed a panel of SOX10-deleted derivatives of A375 cells, and analyzed several single cell edited isolates to provide independent biological replicates. In each of these clones, the absence of SOX10 elevated the level of IRF1 protein (Fig. 3B), confirming an inverse correlation between IRF1 and SOX10 protein levels.

To determine whether there is a direct dependence of p53 on SOX10 and IRF1 proteins (Fig. 3A), we reintroduced wt p53 by transient transfection of A375p53KO and RPMI7951 cells. Transfected cells exhibited p53 protein 24 h and 48 h after transfection, with an increase in IRF-1, while SOX10 remained unchanged in these cells (Fig. 3C, 3D).

We have established that p53-null cells contain lower levels of SOX10 than cells with wt p53. Moreover, p53 activation did not alter SOX10 protein levels (Fig. 3A). To explore the underlying mechanisms further, we searched for p53 response element motifs within the promoter regions, specifically within 1 kilobase upstream of the transcription start site of *SOX10*, *IRF1*, *IRF4*, and *CD274*. These investigations did not provide evidence of such motifs, suggesting that these genes are not directly regulated by p53 at the transcriptional level. Instead, it appears that the presence of p53 is essential for their regulation through other mechanisms.

**Fig. 4:**
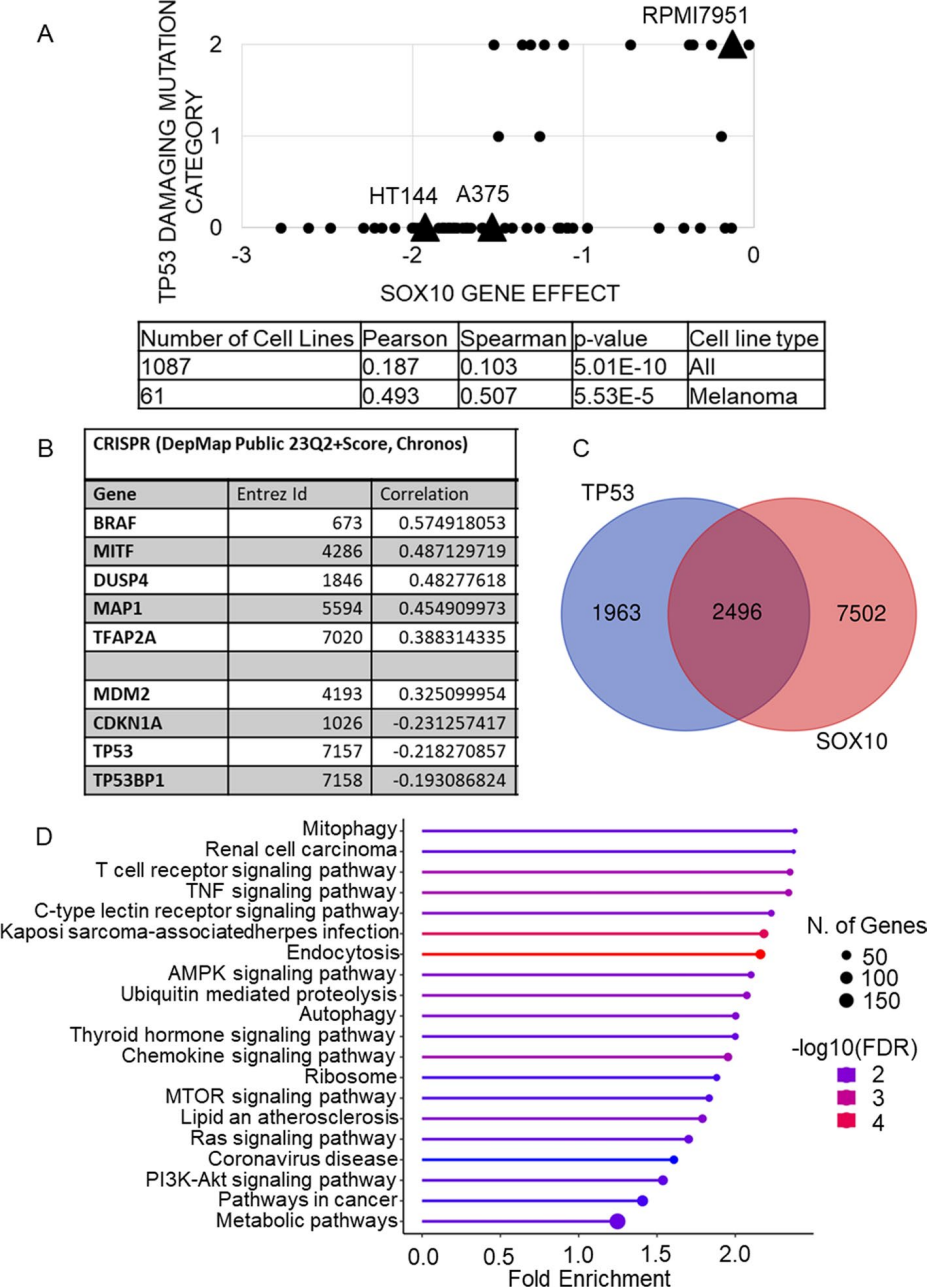
Bioinformatic analysis of SOX10 dependencies on p53 pathway. **A** The dependence of 64 melanoma cell lines for SOX10 gene effect from CRISPR (DepMap Public 23Q2 + Score, Chronos) on the *x*-axis (0 is equivalent to a gene that is not essential, less than −1 corresponds to essential genes) to *TP53* mutation status on the *y*-axis (0 is no mutation, 1 is other mutations, 2 is damaging mutation as defined by DepMap portal). A375, HT144, and RPMI7951 are highlighted. The table shows Pearson and Spearman correlation coefficients for all cell lines and for melanoma cell lines. **B** The co-dependent genes for SOX10 from CRISPR (DepMap Public 23Q2 + Score, Chronos), showing the top five genes and the p53-related genes among the top 100 co-dependent genes, with their Pearson correlation coefficients indicating the strength of co-dependency between SOX10 and the indicated genes. **C** Venn diagram showing genes significantly co-expressed between *TP53* and *SOX10* (in intersection). The mRNA data were downloaded from cbioportal.org. **D** KEGG pathway enrichment analysis of genes significantly co-expressed with *TP53* and *SOX10.* Visualized by ShinyGo0.77

We searched for possible explanations in DepMap (https://depmap.org/portal/) including cbioportal (https://www.cbioportal.org/), with a focus on melanoma. According to CRISPR (DepMap Public 23Q2 + Score, Chronos), *SOX10* is classified as an essential gene in melanoma (gene score effect less than −1). There is a notable correlation between *TP53* damaging mutations and *SOX10* as an essential gene in cancer (Fig. 4A). The correlation can be explained by a cell line bearing a *TP53* damaging mutation, with it being less probable that *SOX10* will be an essential gene. In other words, when *TP53* is wt, cell lines need SOX10 to survive. These findings emphasize the significance of p53 status in influencing SOX10. Furthermore, we searched for genes that exhibit the highest degree of co-dependence with SOX10 using data from CRISPR (DepMap Public 23Q2 + Score, Chronos), and identified genes associated with p53 pathway signaling (Fig. 4B).

SOX10 and p53 are transcription factors with numerous target genes that regulate multiple signaling pathways. To identify potential regulatory relationships and functional associations between genes in a biological context, we examined genes that exhibit significant co-expression (Fig. 4C). Several pathways are enriched using KEGG, with considerable representation of immunology-related processes, autophagy, and metabolic signaling (Fig. 4D). Furthermore, we analyzed genes co-expressed between *SOX10*, *TP53,* and *IRF1*, and divided significantly co-expressed genes into several categories. We chose genes that demonstrate a reverse co-expression pattern of *IRF1* compared with *TP53* and *SOX10* (Fig. S6A). This decision is grounded in experimental data, which indicates that these proteins are expressed in cancer cell lines where IRF1 is downregulated, while IRF1 is upregulated in cell lines where SOX10 and p53 are decreased or absent (Fig. 4A). Similar amounts of co-expressed genes were found between these two conditions (Fig. S6A). Pathway analysis identified high enrichment of immunological processes in IRF1 positively correlated and TP53 and SOX10 negatively correlated groups (IRF1+ , TP53−, SOX10−) (Fig. S6B). The other group of genes (IRF1−, TP53+ , SOX10+) are enriched in only three pathways, none of which are involved in immune regulation (Fig. S6C). These results suggest that p53 cross-talks to SOX10 to regulate common pathways, and their insufficiency affects immunological processes in melanoma.

### Co-dependence in melanoma cell line panel and TCGA datasets based on p53 status.

To address how common the observed dependencies are, we examined nine melanoma cell lines, five bearing wt p53 and four bearing mutant p53 (Table S1), and performed WB to measure key biomarkers (Fig. S7A). We identified correlations of PD-L1 and IRF1 levels across all wt and mutant p53 cell lines. Other factors do not show dependence on each other. IRF4 levels are different from those of SOX10. SOX10 levels depend on p53 presence but not mutation, confirming that SOX10 is not under p53 transcriptional control, but another mechanism is involved and p53 loss is necessary for SOX10 expression (found only in RPMI7951 cells across p53 mutant cell lines). However, it is possible that SOX10 ablation would result in upregulation of IRF1 and PD-L1 proteins in cells with wt p53 and endogenous SOX10 protein.

To gain insight into more relevant biological samples, we explored data from patients with melanoma using differential expression analysis of samples bearing wt *TP53* and those bearing hot-spot mutations in *TP53*. We did not find any significant differences in the mRNA levels of the selected set of genes (Fig. S7B). We then performed co-expression analysis using only wt *TP53* samples to identify factors that associate with functional p53 (Fig. S7C). We identified that *JAK1*, *JAK2*, and *STAT1* show positive correlations with *TP53*, implying that these genes are likely under the regulatory influence of p53.

### p53 activation cross-talks to IFNγ in stimulated cells.

**Fig. 5:**
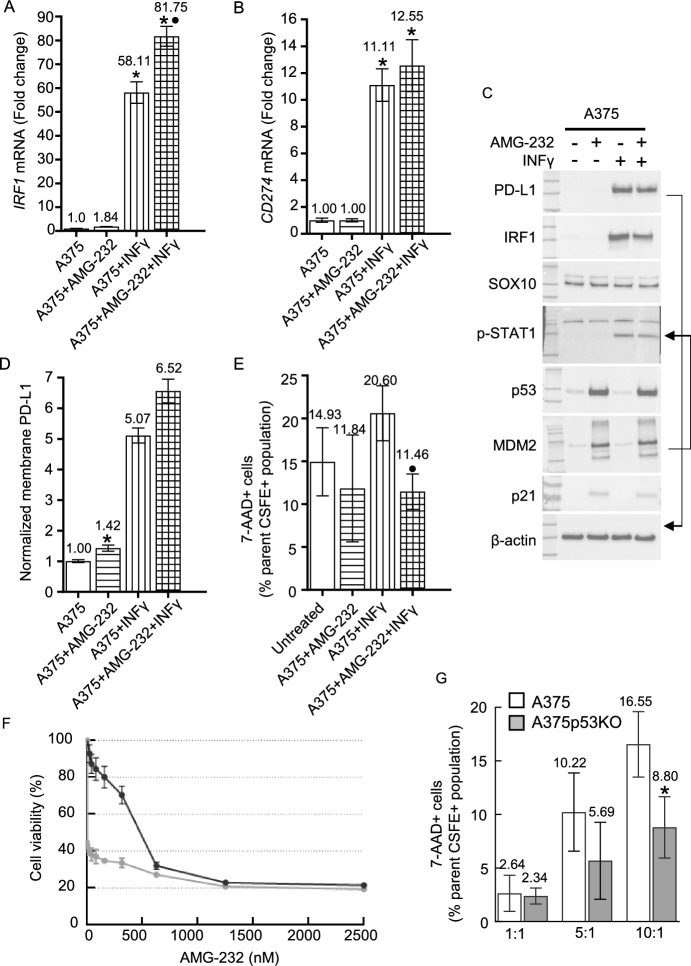
Cross-talk of p53 activation and INFγ stimulation. **A**, **B** RT-qPCR of *IRF1* (A) and *CD274* (B) in A375 cells treated with INFγ (100 ng/mL, horizontally striped column) or AMG-232 (1 μM, vertically striped column) or their combination (hatched column) for 24 h. mRNA levels were normalized to *ACTB* mRNA (*n* = 3 biological replicates, each with technical triplicates). Relative levels are shown as fold change ± SEM compared with untreated A375 (*P < 0.01) or cells treated only with IFNγ and its combination with AMG-232 (*P* < 0.01). **C** Western blots of A375 cells treated as in (**A**) and (**B**). PD-L1, IRF1, SOX10, p-STAT1, p53, MDM2, and β-actin (as loading control) have apparent molecular weights of 55, 55, 55, 80, 90, and 42 kDa, respectively. Stripped and re-probed blots are indicated by arrows. **D** Flow cytometric analysis of membrane PD-L1 in A375 cells treated as in (**A**) and (**B**). PD-L1 levels were normalized to untreated A375 cells and are presented as the mean fluorescence intensity of replicates ± SD (*n* = 3), *P < 0.01. **E** NK cell-mediated cytotoxicity assay in A375 cells treated as in (**A**) and (**B**). Co-culture was performed at 10:1 (NK: tumor cell) ratio. The percentage of dead cells (7-AAD+) in the tumor cell population (CFSE+) is shown. Data are presented as mean ± SEM (*n* = 3 biological replicates) for cells treated only with IFNγ compared with its combination with AMG-232 (*P* < 0.01). **F** Resazurin viability assay of A375 cells treated with only AMG-232 (black line) at the indicated concentrations, or in combination with 100 ng/ml INFγ (grey line) for 72 h. Each sample was measured in five replicates. **G** NK cell-mediated cytotoxicity in A375 (white columns) and A375p53KO (grey columns) at the indicated NK: tumor cell ratios. The percentage of dead tumor cells (7-AAD+ and CFSE+) is shown. Data are presented as mean ± SEM for A375 compared with A375p53KO cells at 10:1 NK: tumor cell ratio (*n* = 3 biological replicates), *P < 0.05

The tumor microenvironment is a complex milieu, and many immunological molecules act on tumor cells. To understand the effect of p53 activation in this more complex view, we used IFNγ as one of the main extracellular regulators of IRF1/PD-L1. We used IFNγ treatment alone or in combination with AMG-232 for 24 h in the A375 cell line. RT-qPCR was employed to assess alterations in *IRF1* and *CD274* mRNA levels (Fig. 5A, B). IFNγ treatment caused significant changes and dual treatment with AMG-232 increased *IRF1* mRNA levels compared with IFNγ treated cells (Fig. 5A). Dual treatment also increased *CD274* mRNA, although this change was not significant compared with cells treated only with IFNγ (Fig. 5B). As previously described, AMG-232 did not appreciably change total PD-L1, IRF1, or SOX10 protein levels, while p53 and MDM2 were increased (Fig. 5C).

IFNγ treatment increased the levels of PD-L1 and IRF1 (as expected) and increased the levels of phosphorylated STAT1 (as expected). There were no changes in SOX10, p53, or MDM2 after IFNγ stimulation. Co-treatment with IFNγ and AMG-232 has opposing effects on PD-L1 and IRF1 protein levels, where small decreases were seen. IFNγ and AMG-232 co-treatment had little effect on p53 and MDM2 compared with AMG-232 alone (Fig. 5C). We next examined the effect of co-treatment on PD-L1 membrane levels. There is an approximately fivefold increase in membrane PD-L1 after IFNγ stimulation alone, reaching a 6.6-fold increase in combination with p53 activation (Fig. 5D).

IFNγ is known for its antiproliferative effects, and we therefore examined the effect of co-treatment with AMG-232. Cells were exposed to AMG-232 (0–2.5 μM) alone and in combination with a constant IFNγ concentration (Fig. 5F). IFNγ alone reduced cell viability to 40%, aligning with the known effect of IFNγ on melanoma cells [51]. IFNγ did not further increase cell death when used in combination treatment with AMG-232 at concentrations that maximally effect cell viability, implying that the mechanism of action of AMG-232 dominates over the effects of IFNγ at higher concentrations, rendering any additional impact from IFNγ negligible.

To assess the influence of *TP53* status on melanoma cells within the immune microenvironment, we used co-culture of tumor cells with a stable cell line of NK cells, NK-92. NK cells induce cell death in melanoma cells and their effectiveness was shown to be influenced by PD-L1 protein levels on tumor cells [52, 53]. In our experiments, we used the NK-92 cell line expressing PD-1 [54]. NK-92 cells induced cell death in A375 cells at specific co-cultivation ratios (Fig. 5G). For A375p53KO cells that express higher basal levels of PD-L1 than parental A375 cells (Fig. 1A, B), we observed a diminished cytotoxic activity toward tumor cells (Fig. 5G). These results demonstrate that p53 loss influences the immune response in contact with immune cells by virtue of PD-L1 protein regulation, suggesting an influence of p53 status on immune evasion.

To examine the possible effect of MDM2 inhibition in IFNγ-stimulated cells, we used the NK cell cytotoxicity assay (Fig. 5E) and pretreated A375 cells with AMG-232, IFNγ, or AMG-232 plus IFNγ for 16 h. NK cell-mediated cytotoxicity against A375 cells without treatment led to approximately 15% cell death. AMG-232 alone had no significant effect on cell survival, whereas IFNγ increased cell death to above 20%. However, tumor cell death is decreased after co-treatment with AMG-232 and IFNγ. This result is similar to those obtained from cell viability assays, where AMG-232 dominates over the effects of IFNγ.

## Discussion

The tumor suppressor p53 has been studied for its role in cancer for more than 40 years. In addition to functions in cell cycle arrest, DNA repair, apoptosis, and metabolism, its role in immune surveillance is now emerging [21, 55–57]. p53 is involved in MHC class I and II presentation [15, 58], interleukin production [59, 60], interferon signaling [61], inflammasome formation [62], T-cell recognition [63], NK-cell recognition [64, 65], and endogenous retrovirus production [19]. It has also been shown that p53 influences PD-L1 expression in various cancer types. This regulation is cancer-type dependent, and in melanoma does not involve the same regulatory nodes as in lung cancer [30, 66], highlighting the importance of studying regulation in a relevant and context-specific manner.

We have previously shown the dependence of PD-L1 on p53 status and activation in human colon cancer HCT116 cells compared with an isogenic HCT116 p53−/− cell line, and in a melanoma cancer cell line A375 and its isogenic p53KO cell line [25]. Interestingly, loss of p53 and p53 activation increase membrane PD-L1, but the mechanism behind this regulation has not been clarified. We confirmed that p53 loss influenced total and membrane PD-L1 levels, and that these differences correlate with *CD274* mRNA levels (Fig. 1). When investigating IRF1 protein levels as a direct transcriptional regulator of PD-L1, we observed a significant difference between wt p53 and p53-null cells, consistent with PD-L1 regulation by p53. However, unlike IRF1 protein, *IRF1* mRNA levels are not altered in A375p53KO cells, excluding transcriptional control of *IRF1* on the basis of p53 status (Fig. 2). Thiem et al. [30] showed that p53 status is predictive for PD-L1 expression in melanoma upon IFNγ treatment, and this regulation was explained by the involvement of JAK-STAT signaling. We tested this hypothesis in our cell line models but did not find dependence of p53 status on JAK2 expression, nor STAT1 activation (Fig. S5 and S7).

Therefore, we looked at a recently described connection between IRF1 and its regulation by SOX10 and IRF4 [32]. SOX10 is an essential transcription factor in neural crest cells, and is highly expressed in melanoma [67], contributing to its tumorous behavior [68]. The involvement of SOX10 in melanoma immunogenicity has been studied extensively, showing that its loss sensitizes tumor cells to T-cell mediated killing [69], and it is a direct transcription factor for immune molecules such as CEACAM1 [70, 71] and IRF4 [32]. In our experiments, in addition to the connection between SOX10 and IRF1, we discovered that SOX10 is likely dependent on p53 status, and we identified an inverse correlation between IRF1 protein levels and SOX10 and its impact on the IRF1/SOX10 regulatory axis. Unfortunately, we could not confirm IRF4 involvement in this regulatory axis in the A375 cell line, as this protein was not detectable. IRF4 is highly expressed in HT144 cells, but was not correlated with SOX10 protein in the p53-null cell line RPMI7951. Interestingly, IRF4 showed dependence on p53 activation in HT144 cells (Fig. 3A).

A recent study showed that SOX10 deficiency correlates with resistance to immunotherapy and targeted therapies, demonstrating a role for SOX10 in an immune regulatory network [72]. Because p53 is also an important regulator of immunity, we identified genes that are co-expressed with *SOX10* and *TP53*, revealing common pathways influenced by these transcription factors (Fig. 4C), including multiple immune-related pathways (Fig. 4D). In additional analysis, we stratified samples on the basis of the co-expression pattern with *SOX10*, *TP53*, and *IRF1*. Interestingly, immune pathways were identified only in *IRF1* positive co-expressed genes, while *SOX10* and *TP53* negatively correlated with the levels of immune pathway genes, suggesting that SOX10 deficiency and p53 deficiency are related to IRF1 network activation (Fig. S6). The dependence of PD-L1 on SOX10 was shown in A375 cells, where SOX10 overexpression increased PD-L1 and SOX10 knockdown decreased PD-L1 levels [73]. On the contrary, in our experiments, loss of SOX10 expression based on p53 loss correlated with increased PD-L1 and IRF1 protein levels.

In our experiments, p53 activation does not influence SOX10 protein levels and there is no p53 response element in the *SOX10* promoter, arguing against *SOX10* being a direct transcriptional target of p53. In our melanoma cell line panel, there is no correlation between *TP53* gene status and SOX10 protein level, which points to p53 presence/absence and not mutation influences SOX10 protein levels. Different p53 mutation types have different impacts on function. For example, hot-spot mutations cause insufficiency in binding target DNA sequences. However, p53 exerts multiple functions, of which only some may be lost by the specific mutation, and many gain of function p53 mutations have also been described [74]. The hypothesis that p53 mutations do not always cause changes of SOX10 activity is strengthened by our observations that only damaging *TP53* mutations correlate with a weaker essentiality of SOX10 in melanoma.

One intriguing finding of our research is that reintroduction of p53 to p53-null cell lines is not sufficient to restore either SOX10 or IRF1 levels. p53 loss may have irreversible effects on the cell because core DNA repair and genome integrity pathways are impaired [75–77]. Moreover, we have previously described the evolution of mutations after p53 deletion in A375 cells [78], and we can speculate that genetic changes may be responsible for SOX10 alteration. However, a problem with p53 transfection is that the high levels of exogenous wt p53 cause growth arrest, while cells with wt p53 at endogenous levels can grow. Thus, if we had an assay to re-introduce p53 at physiologically relevant levels and with appropriate negative feedback where cells continue to grow, SOX10 would be restored. To overcome this issue, we would need to use gene editing to restore the wt p53 sequence to the p53 mutant genes in these p53 cell models. Such “diploid” wt p53 cells could then be examined to determine whether SOX10 protein levels are restored or whether loss of p53 creates an irreversible loss of SOX10 expression.

Surprisingly, p53 activation as well as p53 loss led to increased membrane exposition of PD-L1, demonstrating that two different pathways exploit p53 status to regulate PD-L1, and that a complicated mechanism of crosstalk between these pathways is involved. Drugs targeting the p53-MDM2 interaction by binding to the p53-binding pocket of MDM2 have been widely used in p53 research and many are evaluated in clinical trials, even in combination with immunotherapies [22]. Several groups have observed the effect of p53 activation on PD-L1. Specifically, p53 activation in a melanoma model led to increased membrane PD-L1 levels [25]. By studying p53 activation with the specific MDM2 inhibitor AMG-232, we characterized the impact on IFNγ signaling in a broader view. p53 activation leads to higher PD-L1 protein membrane expression, but the total protein levels and mRNA levels remain static. Further, p53 activation leads to transcriptional activation of *IRF1*, but this is not seen at the IRF1 protein level.

p53 activation induces *MDM2* transcription, and IRF1 was described as a proteasomal target of MDM2 [44, 45]. However, we did not find differences in IRF1 protein half-life or the influence of proteasome inhibition on IRF1 protein levels (Fig. 3). Activation of MDM2 has opposing effects on p53, and these two events can result in undetectable changes under normal conditions. Often, such changes are more visible in a specific condition, as with NFκB activation by p53 or the effect of MDM2 on p53 stability under stress conditions [46, 79]. Therefore, we investigated the effect of the MDM2 inhibitor in cells stimulated by IFNγ.

IFNγ is released by immune cells in the tumor microenvironment and tumor cells respond by activation of JAK-STAT signaling, leading to IRF1 activation. Indeed, we identified increased *IRF1* and *CD274* mRNA levels after exposure of melanoma cells to IFNγ, correlating with increased membrane PD-L1. A peculiar finding is that IRF1 protein levels are decreased by combination treatment. We speculate that the dual treatment increases IRF1 levels that activate its target genes efficiently, but its half-life is decreased, either by proteasomal degradation or by other mechanisms. There is no evidence yet of MDM2 acting on IRF1 at endogenous levels, and future investigations under these conditions will be required to clarify these issues.

## Conclusions

In this study we reported two pathways whereby p53 impacts on the IRF1-PD-L1 axis in melanoma. In one pathway, there is an impact of *TP53* gene status on the IRF1-PD-L1 axis, suggesting that p53 protein loss but not mutation is linked to IRF1-PD-L1 signaling, and that these are correlated to changes in SOX10. The mutual influence of SOX10 and p53 is underlined by SOX10 essentiality in melanoma with wt p53 status. In the second pathway, p53 activation (using MDM2 inhibitors rather than cellular stress) resulted in multiple changes in JAK-STAT-IRF1-PD-L1 signaling, resulting in one masking the other. The stimulation of tumor cells by IFNγ boosts some of these effects. Therefore, investigating a range of specific conditions should be the focus of future research to uncover the impact of these two pathways on IRF1-PD-L1 regulation to identify a clinical benefit from p53-targeted therapies.

## Supplementary Information

Additional file 1: **Figure S1** Gating strategy for analysis of NK cytotoxicity assay (BD FACSuite). **(A)** Initial P1 gate was set in FSC/SSC plot to deplete debris and larger clusters of cells. **(B)** Individual cells were selected in gate P3 omitting cell doublets. **(C)** CFSE+ (tumor) cells are distinguished from NK cells by fluorescence intensity in the FITC channel (gate P4) within the P3 population. **(D)** Positive events in the 7-AAD channel are considered as dead cells. P6 gate includes dead cells within P4 parent population (7-AAD+ events from the CFSE+ population). All gate settings were adjusted on the basis of single cell line samples (A375 only, NK-92 only) and unstained control samples. **Figure S2** Representative cytometric plot of untreated A375 (black solid lines) and A375p53KO (red solid lines) or the same cells treated with 1 μM AMG-232 for 24 h (dotted lines). Dot plots corresponding to both variants are shown below. **Figure S3** **(A)** Western blots of PD-L1 (55 kDa) and β-actin (42 kDa) as loading control for HT144 and RPMI7951 cells treated with/without treatment of 1 μM AMG-232 for 24 h. Relative PD-L1 densitometry measurements were normalized to β-actin are indicated. **(B)** Relative PD-L1 densitometry measurements from WB shown in (A) presented as the mean of replicates ± SD (*n* = 3). **Figure S4** Western blots in A375 and A375p53KO cells with or without 1 μM AMG-232 for 24 h and additionally treated with 20 μM MG-132 or CHX (100 μg/ml) for the last 4 h or 2 h before harvesting, respectively. IRF1, PD-L1, SOX10, MDM2, p53, p21, and β-actin (as loading control) have apparent molecular weights of 55, 55, 55, 90, 53, 21, and 42 kDa, respectively. **Figure S5** Western blots for the indicated proteins in A375, and A375p53KO cell lines with/without treatment of 1 μM AMG-232 for 24 h. STAT1, p-STAT1, NFκB, p-NFκB, JAK2, JAK1, and β-actin (as loading control) have apparent molecular weights of 80, 80, 120, 135, 65, 65, and 42 kDa, respectively. **Figure 6** **(A)** Venn diagram showing genes significantly co-expressed between *TP53*, *SOX10*, and *IRF1* (in intersection) and the division of genes from intersection based on positive (marked as + for given gene) or negative (marked as − for given gene) correlation. The mRNA data used in this analysis were downloaded from cbioportal.org. for melanoma panel TCGA Firehose legacy. **(B)** KEGG Pathway enrichment analysis of 464 significantly co-expressed genes for group: IRF1+, TP53− and SOX10− from previous Venn diagram visualized by ShinyGo0.77. **(C)** KEGG pathway enrichment analysis of 406 significantly co-expressed genes from group IRF1−, TP53+, SOX10+ from previous Venn diagram visualized by ShinyGo0.77. **Figure S7 (A) **Western blots for the indicated proteins in melanoma cell lines bearing wt p53 or p53-null. PD-L1, IRF1, IRF4, SOX10, JAK1, JAK2, STAT1, p53, and β-actin (as loading control) have apparent molecular weights of 55, 55, 55, 55, 135, 120, 80, 53, and 42 kDa, respectively. **(B)** The differential mRNA expression analysis of selected genes between wt *TP53* and *TP53* hot-spot mutant patient samples. The data were extracted from cbioportal.org from SKCM data pack (number of samples 472) listed as cohort GDC TCGA Melanoma. Log2ratio shows the fold change between wt and hot-spot mutant group. **(C)** Co-expression analysis of *TP53* mRNA for wt p53 patient samples and selected genes. Pearson correlation coefficient with corresponding *P*-value demonstrating correlation strength is shown. Significantly co-expressed genes are highlighted in bold. **Table S1** Oncogenic mutations in selected melanoma cell lines according to DepMAp. **Table S2** List of antibodies used for WB.

## Data Availability

The data supporting the conclusions of this article are available from the corresponding author on reasonable request.

## Abbreviations

CHX: Cycloheximide
CTLA4: Cytotoxic T-lymphocyte-associated protein 4
IFNγ: Interferon-γ
IRF1: Interferon regulatory factor 1
IRF4: Interferon regulatory factor 4
JAK: Janus kinase
MDM2: Mouse double minute 2 homolog
NFκB: Nuclear factor kappa-light-chain-enhancer of activated B cells
NK: Natural killer
PD-1: Programmed cell death 1
PD-L1: Programmed cell death 1 ligand 1
SOX10: SRY-box transcription factor 10
STAT: Signal transducers and activators of transcription
