# Supplementary material for: Inverse correlation between TP53 gene status and PD-L1 protein levels in a melanoma cell model depends on an IRF1/SOX10 regulatory axis
Source: Cell Mol Biol Lett. 2024 Sep 5;29:117. doi: 10.1186/s11658-024-00637-y (PMC11378555; doi:10.1186/s11658-024-00637-y)
Supplement: Supplementary file 1 — Additional file 1: Figure S1 Gating strategy for analysis of NK cytotoxicity assay (BD FACSuite). (A) Initial P1 gate was set in FSC/SSC plot to deplete debris and larger clusters of cells. (B) Individual cells were selected in gate P3 omitting cell doublets. (C) CFSE+ (tumor) cells are distinguished from NK cells by fluorescence intensity in the FITC channel (gate P4) within the P3 population. (D) Positive events in the 7-AAD channel are considered as dead cells. P6 gate includes dead cells within P4 parent population (7-AAD+ events from the CFSE+ population). All gate settings were adjusted on the basis of single cell line samples (A375 only, NK-92 only) and unstained control samples. Figure S2 Representative cytometric plot of untreated A375 (black solid lines) and A375p53KO (red solid lines) or the same cells treated with 1 μM AMG-232 for 24 h (dotted lines). Dot plots corresponding to both variants are shown below. Figure S3 (A) Western blots of PD-L1 (55 kDa) and β-actin (42 kDa) as loading control for HT144 and RPMI7951 cells treated with/without treatment of 1 μM AMG-232 for 24 h. Relative PD-L1 densitometry measurements were normalized to β-actin are indicated. (B) Relative PD-L1 densitometry measurements from WB shown in (A) presented as the mean of replicates ± SD (n = 3). Figure S4 Western blots in A375 and A375p53KO cells with or without 1 μM AMG-232 for 24 h and additionally treated with 20 μM MG-132 or CHX (100 μg/ml) for the last 4 h or 2 h before harvesting, respectively. IRF1, PD-L1, SOX10, MDM2, p53, p21, and β-actin (as loading control) have apparent molecular weights of 55, 55, 55, 90, 53, 21, and 42 kDa, respectively. Figure S5 Western blots for the indicated proteins in A375, and A375p53KO cell lines with/without treatment of 1 μM AMG-232 for 24 h. STAT1, p-STAT1, NFκB, p-NFκB, JAK2, JAK1, and β-actin (as loading control) have apparent molecular weights of 80, 80, 120, 135, 65, 65, and 42 kDa, respectively. Figure 6 (A) Venn diagram showing ge [file 11658_2024_637_MOESM1_ESM.docx]

**Supplementary data**

**
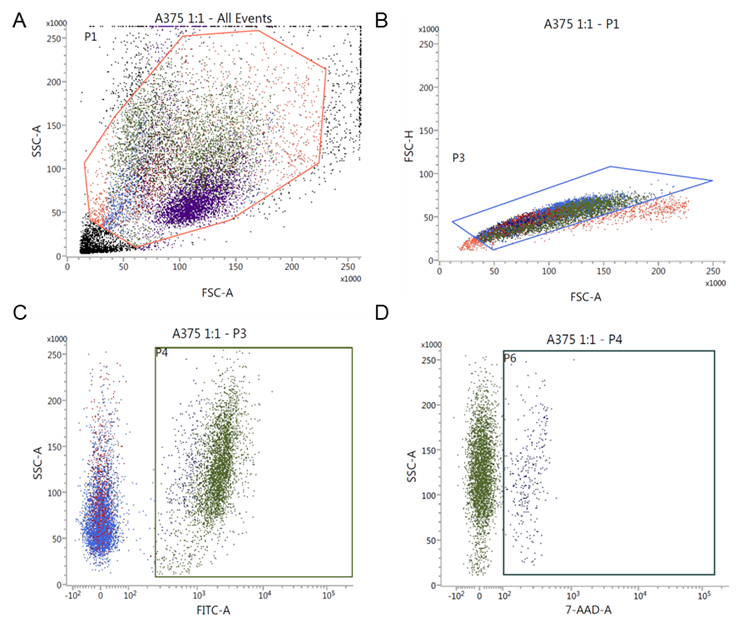
**

**Figure S1** Gating strategy for analysis of NK cytotoxicity assay (BD FACSuite). **(A)** Initial P1 gate was set in FSC/SSC plot to deplete debris and larger clusters of cells. **(B)** Individual cells were selected in gate P3 omitting cell doublets. **(C)** CFSE+ (tumor) cells are

distinguished from NK cells by fluorescence intensity in the FITC channel (gate P4) within the P3 population. **(D)** Positive events in the 7-AAD channel are considered as dead cells. P6 gate includes dead cells within P4 parent population (7-AAD+ events from the CFSE+ population). All gate settings were adjusted on the basis of single cell line samples (A375 only, NK-92 only) and unstained control samples.

**
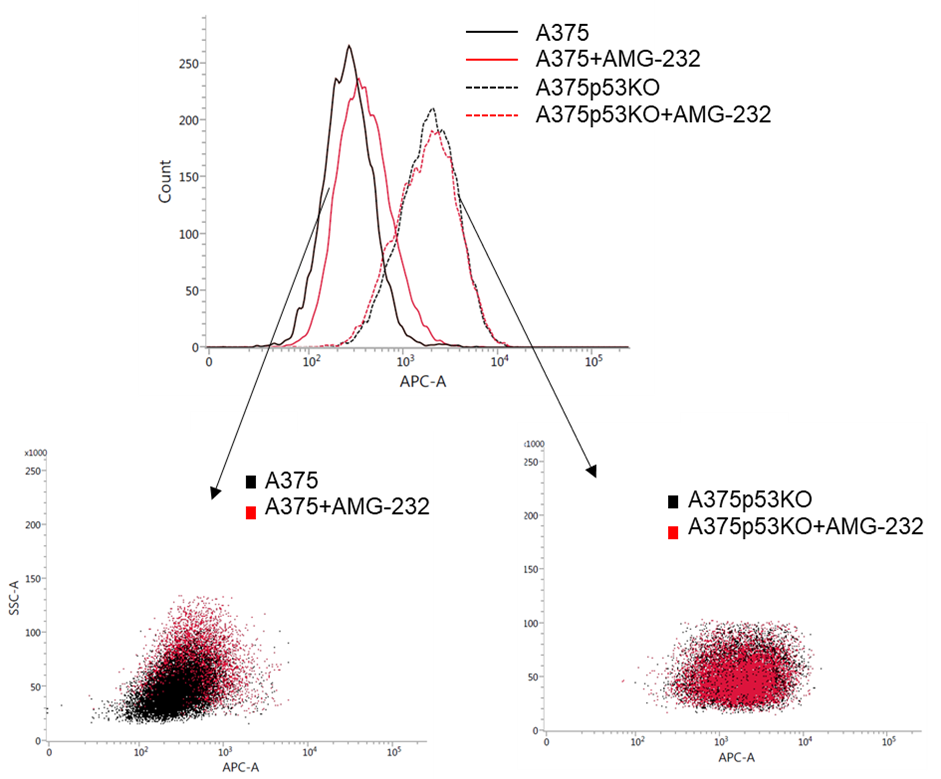
**

**Figure S2** Representative cytometric plot of untreated A375 (black solid lines) and A375p53KO (red solid lines) or the same cells treated with 1 μM AMG-232 for 24 h (dotted lines). Dot plots corresponding to both variants are shown below.

**
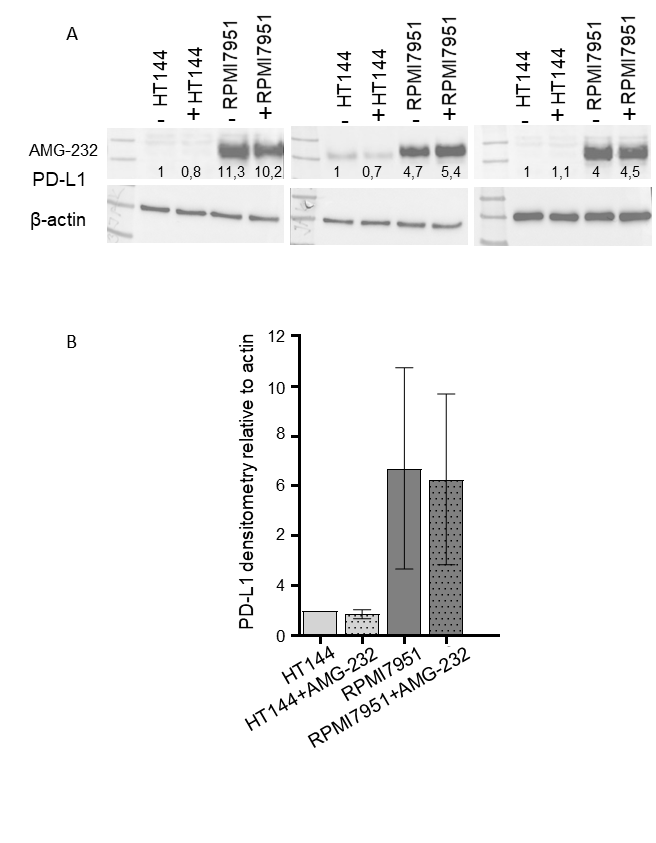
**

**Figure S3** **(A)** Western blots of PD-L1 (55 kDa) and β-actin (42 kDa) as loading control for HT144 and RPMI7951 cells treated with/without treatment of 1 μM AMG-232 for 24 h. Relative PD-L1 densitometry measurements were normalized to β-actin are indicated. **(B)** Relative PD-L1 densitometry measurements from WB shown in (A) presented as the mean of replicates ± SD (n=3).

**
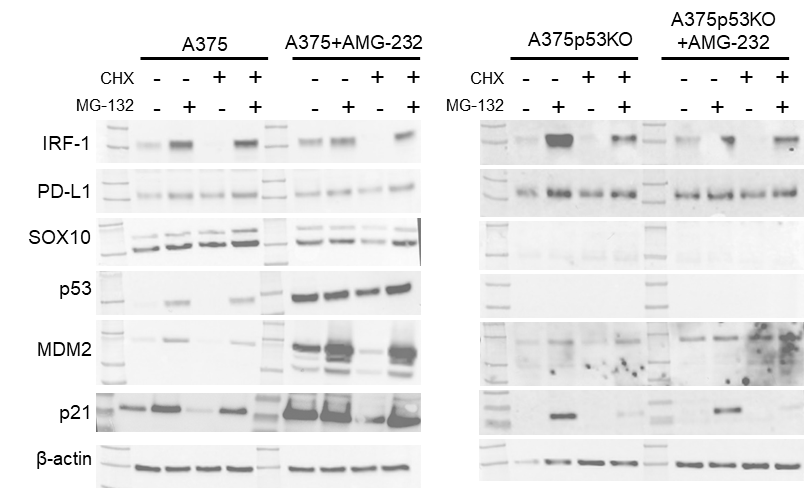
**

**Figure S4** Western blots in A375 and A375p53KO cells with or without 1 μM AMG-232 for 24 h and additionally treated with 20 μM MG-132 or CHX (100 μg/ml) for the last 4 or 2 h before harvesting, respectively. IRF1, PD-L1, SOX10, MDM2, p53, p21 and β-actin (as loading control) have apparent molecular weights of 55, 55, 55, 90, 53, 21 and 42 kDa, respectively.


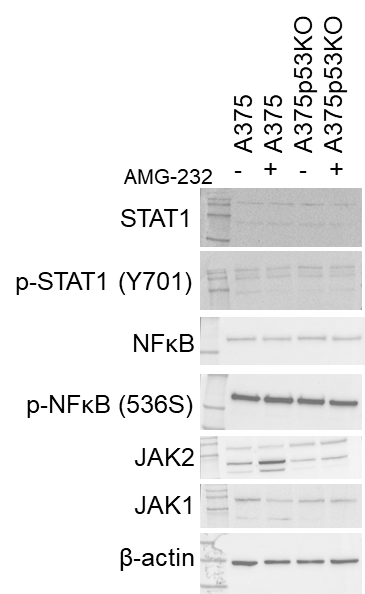


**Figure S5** Western blots for the indicated proteins in A375, and A375p53KO cell lines with/without treatment of 1 μM AMG-232 for 24 h. STAT1, p-STAT1, NFκB, p-NFκB, JAK2, JAK1 and β-actin (as loading control) have apparent molecular weights of 80, 80, 120, 135, 65, 65 and 42 kDa, respectively.


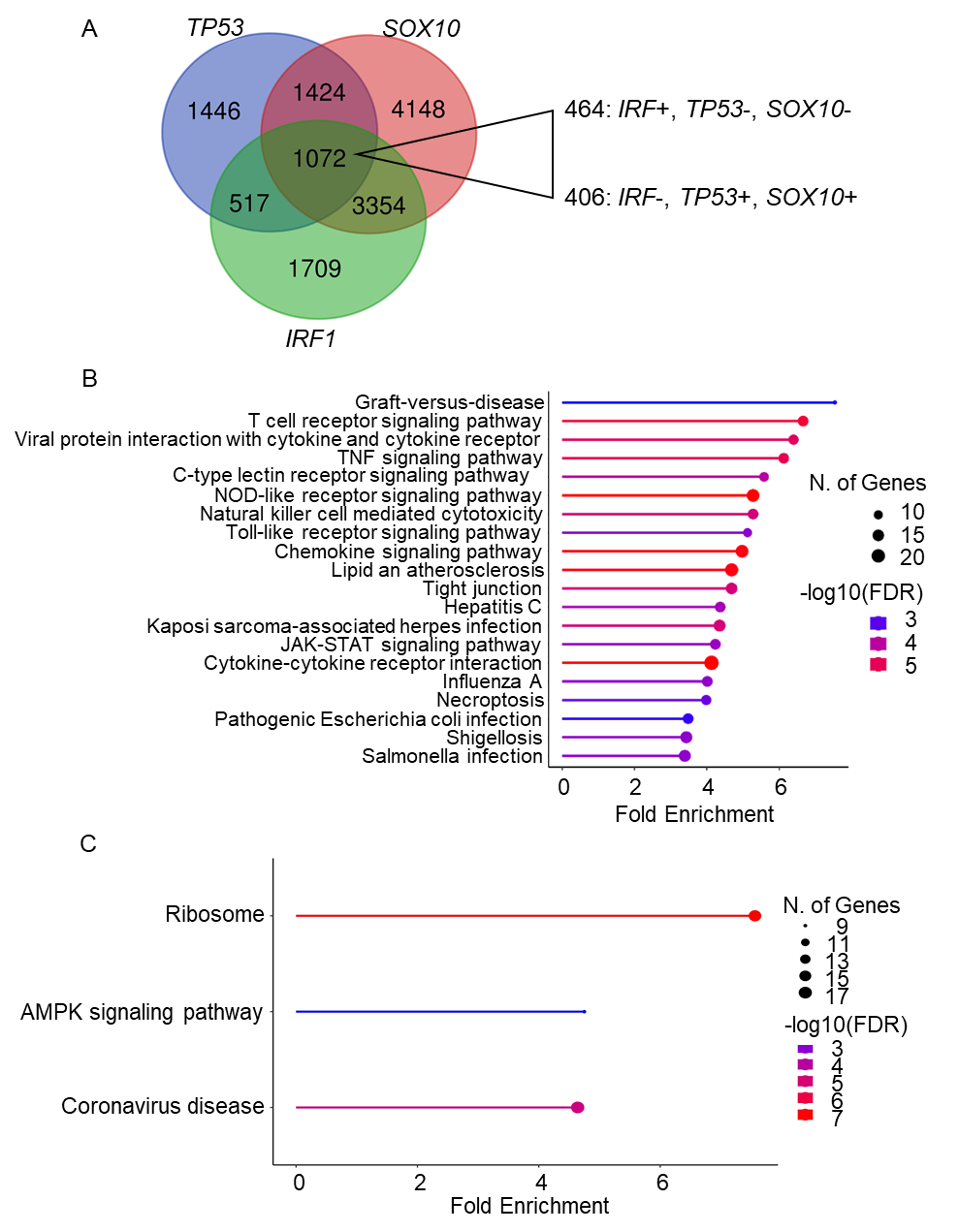


**Figure 6** **(A)** Venn diagram showing genes significantly co-expressed between *TP53*, *SOX10*, and *IRF1* (in intersection) and the division of genes from intersection based on positive (marked as + for given gene) or negative (marked as – for given gene) correlation. The mRNA data used in this analysis were downloaded from cbioportal.org. for melanoma panel TCGA Firehose legacy. **(B)** KEGG Pathway enrichment analysis of 464 significantly co-expressed genes for group: IRF1+, TP53- and SOX10- from previous Venn diagram visualized by ShinyGo0.77. **(C)** KEGG pathway enrichment analysis of 406 significantly co-expressed genes from group IRF1-, TP53+, SOX10+ from previous Venn diagram visualized by ShinyGo0.77.


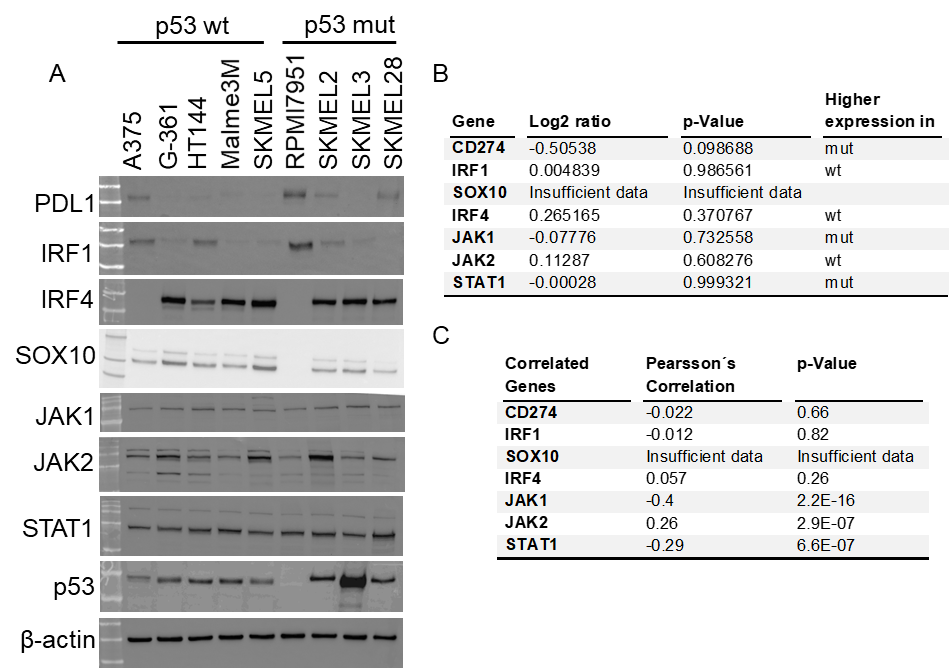


**Figure S7 (A)** Western blots for the indicated proteins in melanoma cell lines bearing wt p53 or p53-null. PD-L1, IRF1, IRF4, SOX10, JAK1, JAK2, STAT1, p53 and β-actin (as loading control) have apparent molecular weights of 55, 55, 55, 55, 135, 120, 80, 53 and 42 kDa, respectively. **(B)** The differential mRNA expression analysis of selected genes between wt *TP53* and *TP53* hot-spot mutant patient samples. The data were extracted from cbioportal.org from SKCM data pack (number of samples 472) listed as cohort GDC TCGA Melanoma. Log2ratio shows the fold change between wt and hot-spot mutant group. **(C)** Co-expression analysis of *TP53* mRNA for wt p53 patient samples and selected genes. Pearson correlation coefficient with corresponding P-value demonstrating correlation strength is shown. Significantly co-expressed genes are highlighted in bold.

| Cell lines | TP53 | BRAF | RAS | CDKN2A |
| --- | --- | --- | --- | --- |
| A375 | wt | V600E | wt | E18*, E10* |
| G361 | wt | V600E | wt | wt |
| HT144 | wt | V600E | wt | wt |
| MALMA3M | wt | V600E | wt | wt |
| SKMEL5 | wt | V600E | wt | wt |
| RPMI7951 | S166* | V600E | wt | L16R |
| SKMEL2 | G245S | wt | P61R | wt |
| SKMEL3 | R267W | V600E | wt | wt |
| SKMEL28 | L145R | V600E | Wt | D23P |

**Table S1** Oncogenic mutations in selected melanoma cell lines according to DepMAp.

| Antibodies | Catalog # | Company |
| --- | --- | --- |
| p53 |  | In house |
| PD-L1 (E1L3N) | 13684 | Cell Signaling Technology |
| PD-L1-APC (MIH1) | 17-5983-42 | Invitrogen |
| SOX10 (5H7L26) | 703439 | Invitrogen |
| IRF1 (D5E4) | 8478 | Cell Signaling technology |
| β-actin (15G5A11/E2) | MA1-140 | Invitrogen |
| IRF-4 ((E8H3S) XP | 62834 | Cell Signaling Technology |
| JAK1 (6G4) | 3344 | Cell Signaling Technology |
| JAK2 (D2E12) XP | 3230 | Cell Signaling Technology |
| MDM2 |  | In house |
| STAT1 | 9172 | Cell signaling technology |
| P-STAT1 (Tyr701) (58D6) | 9176 | Cell Signaling technology |
| NFκB p65 | 3034 | Cell Signaling Technology |
| p-NFκB p65 (Ser536) | 3031 | Cell Signaling Technology |

**Table S2** List of antibodies used for WB.
